# The role of leptin in reproductive dysfunction in patients with varicocele: a systematic review and meta-analysis

**DOI:** 10.3389/fruro.2026.1835856

**Published:** 2026-06-05

**Authors:** Yan Zhang, Yangming Li, Xiaodan Du, Ye Dong, Honglong Jia, Jian Qiu, Bin He, Shengzhuo Liu, Sikui Shen, Yuchun Zhu

**Affiliations:** 1 Department of Urology, Sichuan Provincial Second Traditional Chinese Medicine Hospital, Chengdu, China; 2 Department of Urology, West China Hospital of Sichuan University, Chengdu, China

**Keywords:** infertility, leptin, male, sperm parameters, varicocele

## Abstract

**Background:**

The mechanism of leptin’s role in female infertility has been demonstrated, however, the relationship between it and infertile patients with varicocele has yet to be determined. This study aimed to evaluate role of leptin in reproductive dysfunction in patients with varicocele.

**Methods:**

A computerized literature search was independently conducted by two researchers in PubMed, EMBASE, Web of Science, ScienceDirect, and Cochrane Library up to July 2025. The search terms were as follows: ‘leptin’, ‘varicocele’ and ‘infertility’. The Newcastle-Ottawa Scale was used to assess the quality of the included studies. The meta-analysis was performed using Review Manager version 5.1 and R version 4.0.5.

**Results:**

36 studies were retrieved and 5 studies were included for meta-analysis. We found that the expression of leptin in seminal plasma in infertile patients with varicocele were significantly higher than controls (SMD, 2.72; 95%CI, 1.05-4.39; I2 = 96%, p=0.001). In contrast, the serum leptin levels did not show statistically significant differences (SMD, 1.26; 95%CI -0.73-3.26; I2 = 96%, p=0.21) between patients and controls. Seminal plasma leptin levels had a moderate negative correlation with sperm concentration (r=-0.54; 95%CI, -0.77 - -0.19; p<0.01). Additionally, seminal plasma leptin levels had a strong negative correlation with sperm progressive motility (r=-0.71; 95%CI, -0.93 - -0.09; p<0.01).

**Conclusions:**

The expression of leptin in seminal plasma was significantly higher in infertile patients with varicocele. Seminal plasma leptin levels had a negative correlation with sperm concentration and progressive motility. Leptin in seminal plasma seemed to have a negative effect on the reproductive function of patients with varicocele.

## Introduction

Infertility is a growing public health issue globally, with an estimated prevalence of 10% (1). Male infertility could be caused by a variety of factors, the most prominent of which is varicocele. The incidence of primary infertility with varicocele is reported to be 35–44%, while the incidence of second infertility with varicocele is up to 81% (2).

Leptin, encoded by the obese (ob) gene, is a 16-kDa non-glycosylated peptide hormone. Leptin is primarily synthesized and secreted by white adipose tissue (3). And small amount of it are also secreted in a variety of tissue including anterior pituitary gland (4), placenta (5), ovaries and human spermatozoa (6). Leptin is involved in the various physiological activities, including the regulation of food intake, body weight and insulin signaling (7, 8), and the modulation of the hypothalamic - pituitary - thyroid axes, hypothalamic - pituitary - growth hormone axes, hypothalamic - pituitary - gonadal axes and reproduction (9). Meanwhile, normal expression levels of leptin are essential for normal sexual maturation and function. The congenital deficiency of leptin in human was reported to be associated with hypogonadotropic hypogonadism and delayed puberty. While the excessive expression of leptin may have negative effect on the reproductive function. Numbers of studies indicated that the significantly increased levels of leptin in serum were associated with the reproduction dysfunctions in obesity like hypogonadism and decreased sperm parameters (10–12). Various of mechanisms underlying male infertility associated with leptin have been proposed, such as leptin modulation of testicular physiology, leptin resistance and the insufficiency of leptin in hypothalamus (13).

It has been reported that leptin was related to varicocele-induced spermatogenic dysfunction. Studies found that leptin may impact the reproductive function of male rats with varicocele by influencing the hypothalamic-pituitary-gonadal axes (14, 15). However, the evidence for the relationship between leptin and infertility patients with varicocele remains mixed. The purpose of this study was to summarize the current evidence to evaluate the effect of leptin on reproductive dysfunction in patients with varicocele.

## Methods

### Search strategy and selection criteria

A computerized literature search was independently performed by two investigators across PubMed, EMBASE, Web of Science, ScienceDirect, and Cochrane Library from inception up to July 2025, following the Preferred Reporting Items for Systematic Reviews and Meta-Analyses (PRISMA) guidelines (16). Manual screening of the reference lists of all retrieved articles was also performed to identify additional eligible studies.

The literature search was conducted using MeSH terms combined with free-text keywords. The main MeSH terms included: Leptin, Varicocele, and Infertility. Corresponding free-text synonyms were supplemented for each term, and all terms were combined with the Boolean operator AND to retrieve relevant studies.

The selection of eligible studies was performed independently by two reviewers based on established inclusion and exclusion criteria. The study would be included if: (1) it reported seminal or serum leptin levels or correlations between leptin levels and sperm parameters in patients with varicocele and infertility; (2) the study design was a case-control study or cohort study with the control group. Exclusion criteria are the following: (1) animal studies, letters, reviews, and expert opinions were excluded; (2) studies lacking raw data and the authors could not be contacted were excluded; (3) studies without full text or published in a language other than English were also excluded. The third reviewer would be consulted if discrepancies existed.

### Data extraction and quality assessment

Two researchers independently collected data from the included studies according to a standard extraction developed at the beginning of the study. The characteristics of the included studies would be collected, including study design, comparison, patient characteristics, and results related to leptin. The quality assessments of the included studies were independently performed by two researchers using the Newcastle-Ottawa Scale (NOS) (17). The third reviewer would be required to make final settlement if necessary.

### Definition

The Pearson correlation coefficient (r value) was used to assess the correlation between leptin level and semen analysis results. Typically, the strength of correlation of the variables was determined by the range of absolute values of r: 0.8-1.0 for very strong correlation, 0.6-0.8 for strong correlation, 0.4-0.6 for moderate correlation, 0.2-0.4 for weak correlation, and 0.0-0.2 for very weak or no correlation. A negative value of r indicated that the leptin level was negatively correlated with sperm parameters.

### Statistical analysis

We summarized the collected data according to the Cochrane Handbook for Systematic Reviews of Interventions (version 5.1.0; The Cochrane Collaboration). The meta-analysis of leptin levels was conducted via Review Manager version 5.1 (The Nordic Cochrane Center, Copenhagen, Denmark). P<0.05 was considered statistically significant. Cochran Q test and Higgins I-squared statistics were used to assess the heterogeneity of the included studies. Random effects model was applied if I2>50% and p<0.05; otherwise, the fixed effects model was utilized. Sensitivity analysis will be conducted to determine if high heterogeneity existed by omitting each study. The summary r was calculated via R version 4.0.5 (R Foundation for Statistical Computing, Vienna, Austria) using the “metacor” package. The Spearman correlation coefficients (sr value) were converted into Pearson correlation coefficients according to the study of Rupinski and Dunlap (18).

## Results

### Literature search results

**Figure 1:**
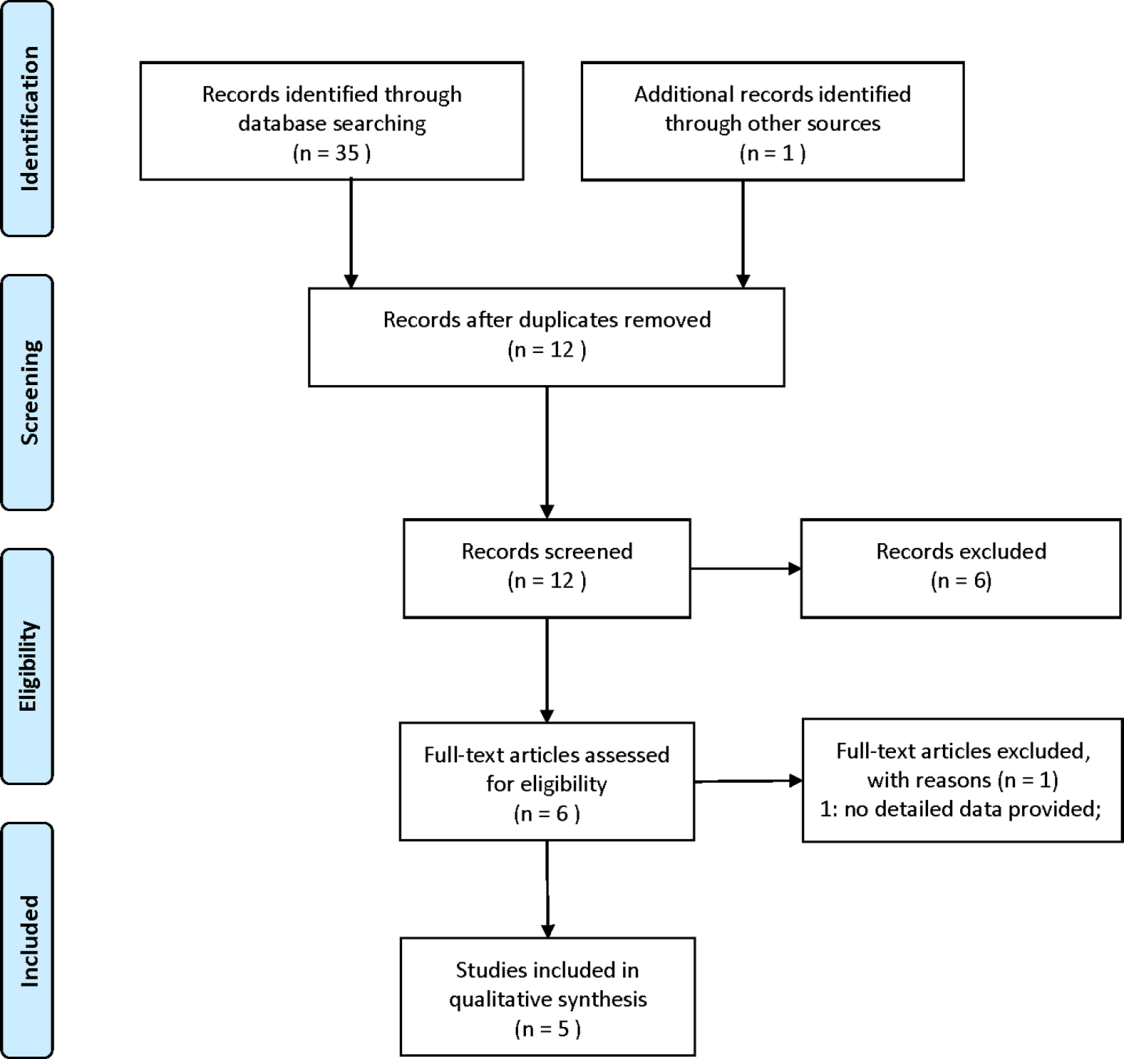
Flow diagram of the search strategy and study selection.

A total of 36 studies were retrieved and six studies were included after removal of duplicates and reading abstract. Finally, 5 studies (14, 19–22) were ultimately identified for meta-analysis with 1 study excluded for lacking of sufficient data (detailed in **Figure 1**). There are 205 patients diagnosed with varicocele and infertility and 119 subjects as controls. All included studies were case-control studies. Among these studies, all compared the expression of seminal leptin between patients and controls, three compared serum leptin levels, and three reported the results of the correlation analysis between leptin and sperm parameters. The characteristics of the included studies were presented in **Table 1**. The results of the quality assessment of the included studies were detailed in **Supplementary Table 1**.

**Table 1:** Characteristics of the included studies.

Author	Year	Country	Design	Participants (n)	Comparison (n)	Age (years, means ± SD)	BMI (Km/m^2, means ± SD)	Seminal leptin level (ng/ml, means ± SD)	Serum leptin level (ng/ml, means ± SD)	Correlations between seminal leptin and sperm parameters	Correlations between serum leptin and sperm parameters	NOS
Abokallal	2020	Iraq	case-control	varicocele with oligozoospermia (n=13)	normal fertile subjects (n=14)	patients: 25.30 ± 2.88; controls: 31.84 ± 1.60	patients: 27.85 ± 1.30; controls: 26.54 ± 0.91	patients: 0.38 ± 0.07; controls: 0.31 ± 0.02	NA	NA	NA	6
Chen	2008	China	case-control	varicocele with infertility (n=40)	normal fertile subjects (n=25)	NA	patients: 22.22 ± 2.95; controls: 22.66 ± 3.14	patients: 4.26 ± 1.88; controls: 1.74 ± 0.87	patients:6.94 ± 2.03; controls:6.50 ± 1.73	volume: NA; concentration: r=-0.632(p=0.000); progressive motility: r= -0.635(p<0.000); normal morphology: NA	NA	9
El Taieb	2018	Egypt	case-control	varicocele with asthenozoospermia (n=36)	normal fertile subjects (n=30)	patients: 32.06 ± 3.3; controls: 32.27 ± 3.1	patients: 22.94 ± 3.1; controls: 21.61 ± 2.4	patients: 4.16 ± 0.7; controls: 2.12 ± 0.1	patients:13.84 ± 3.7; controls:3.96 ± 0.2	volume: r=-0.32(p=0.004); concentration: r=-0.702(p<0.001); progressive motility: r= -0.929(p<0.001); normal morphology: r= -0.711(p<0.001)	volume: r=-0.372(p=0.001); concentration: r=-0.625(p<0.001); progressive motility: r= -0.87(p<0.001); normal morphology: r= -0.645(p<0.001)	9
Ni	2014	China	case-control	varicocele with infertility (n=42)	normal fertile subjects (n=10)	NA	patients: 23.45 ± 0.57; controls: 21.83 ± 1.36	patients: 3.01 ± 1.23; controls: 1.79 ± 0.42	patients:6.44 ± 0.99; controls:6.39 ± 0.78	volume: NA; concentration: rs=-0.187(p<0.05); progressive motility: rs= -0.234(p<0.01); normal morphology: NA	NA	9
Wang	2014	China	case-control	varicocele with infertility (n=74)	normal fertile subjects (n=40)	patients:29.76 ± 13.18; controls: 28.83 ± 5.35	patients: 22.31 ± 0.85; controls: 22.41 ± 1.28	patients: 3.20 ± 0.31; controls: 1.69 ± 0.11	NA	NA	NA	9

### Leptin levels in patients compared to controls

**Figure 2:**
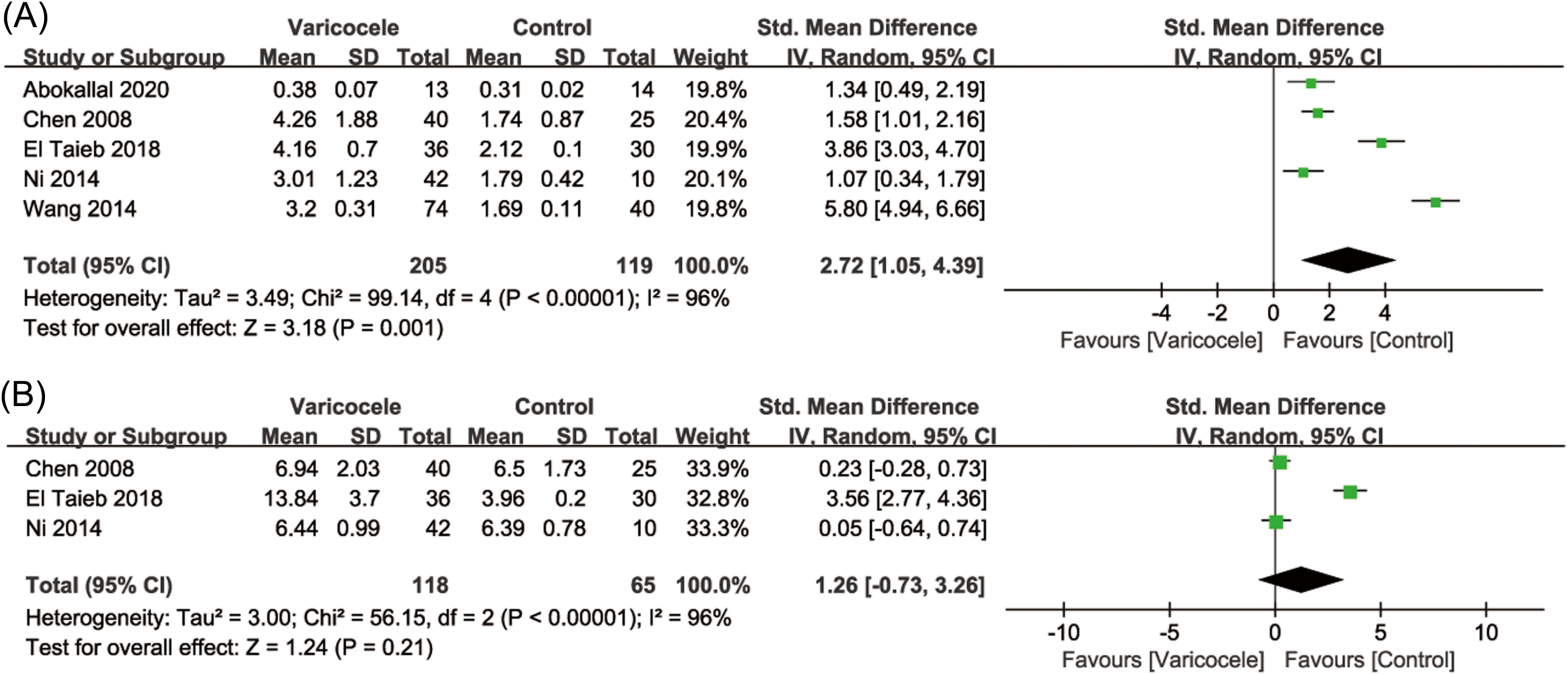
Forest plots showing seminal **(A)** and serum **(B)** leptin levels in patients with varicocele and infertility compared to normal fertile subjects. CI, Confidence interval.

As shown in **Figure 2A**, the expression of seminal leptin in infertility patients with varicocele was significantly higher than that of fertile subjects (SMD, 2.72; 95%CI, 1.05-4.39; I2 = 96%, p=0.001). In contrast, the serum leptin level did not show statistically significant differences (SMD, 1.26; 95%CI -0.73-3.26; I2 = 96%, p=0.21) between patients and controls (**Figure 2B**).

### Correlation between leptin and sperm parameters

**Figure 3:**
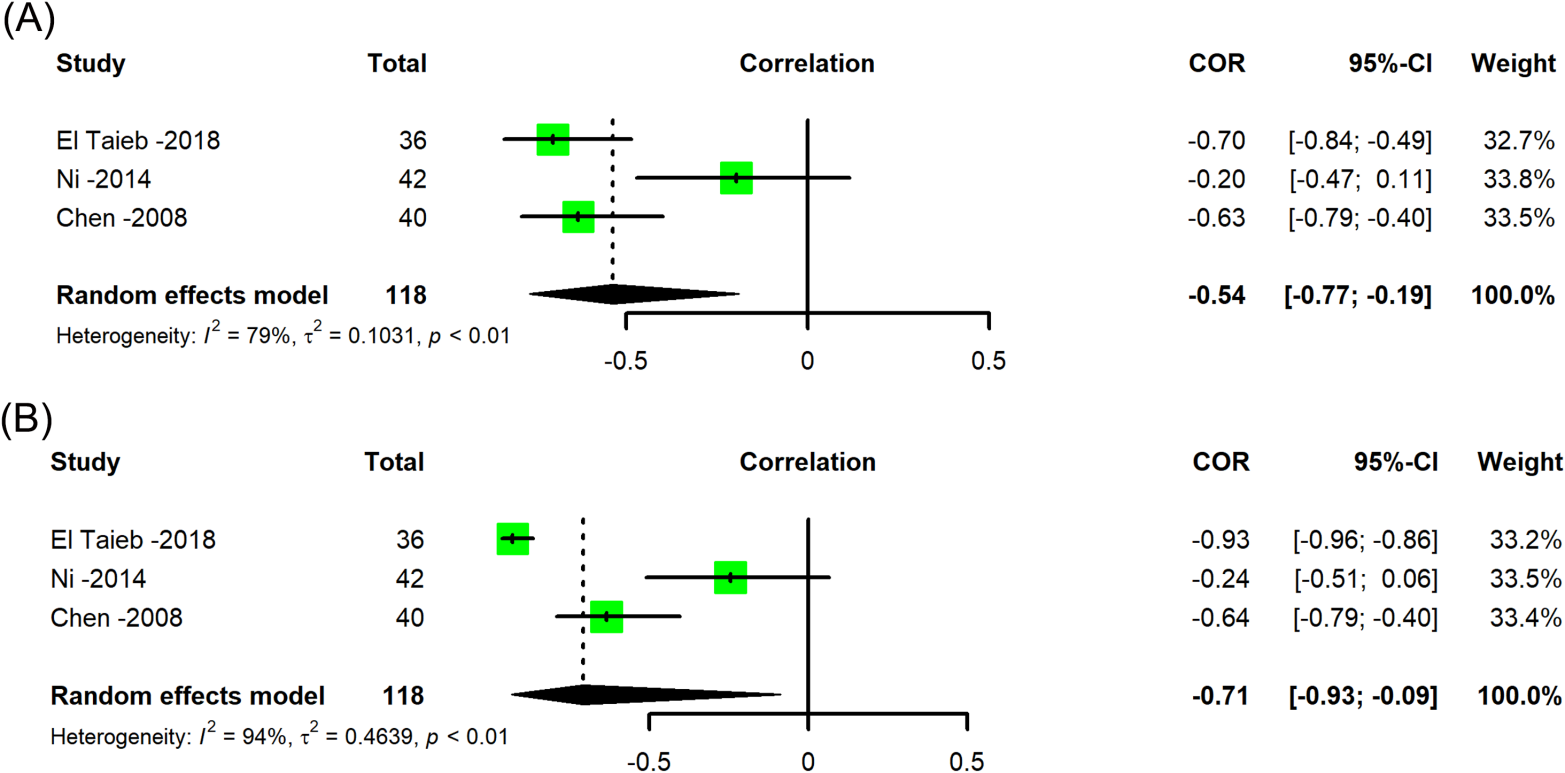
Forest plots showing the summary Pearson correlation coefficient between seminal leptin level and concentration **(A)** and progressive motility **(B)** of sperm. COR, correlation; CI, Confidence interval.

The correlations between the level of seminal leptin and the sperm concentration and the progressive motility were pooled in the meta-analysis. The results indicated that seminal leptin levels had moderate negative correlation with sperm concentration (r=-0.54; 95%CI, -0.77 - -0.19; I2 = 79%, p<0.01) (**Figure 3A**). Furthermore, seminal leptin levels were strongly negative correlation with progressive motility of sperm (r=-0.71; 95%CI, -0.93 - -0.09; I2 = 94%, p<0.01) (**Figure 3B**).

### Sensitivity analysis

Sensitivity analysis suggested that no significant change of the pooled results for all outcomes was observed, excepted for the correlations between seminal leptin level and concentration and progressive motility of sperm (**Supplementary Figure 1**).

## Discussion

In this study, we found that seminal plasma leptin levels were significantly higher in infertile patients with varicocele compared to fertile controls. However, no statistically significant difference in the serum was observed. Furthermore, the expression of leptin in seminal plasma had a negative correlation with sperm concentration and progressive motility. This suggested that the increase of leptin in seminal plasma may be related to the development of human varicocele-induced infertility. And the high leptin levels in seminal plasma seemed to be involved in the sperm dysfunction.

In the study of El Taieb et al. (21), the expression levels of leptin in serum and seminal plasma in infertile patients with varicocele were significantly higher than controls. While no significant increase of leptin in serum was observed according to the study of Zorn et al. (13). In our study, the levels of leptin in serum were not significantly increased but over expressed in seminal plasma. There is no significant difference in serum leptin levels, probably because it is regulated by the balance between expenditure and intake (6, 23). The high seminal plasma leptin levels in infertile patients with varicocele suggesting that the leptin may regulate their own metabolism independently of circulating leptin.

The exact mechanism of the effect of increased seminal plasma leptin on sperm parameters remains uncertain. Expression of leptin and its receptor were found in the mid-section of uninseminated human sperm and the caudal region of the sperm, respectively. Interestingly, after insemination, leptin expression in sperm decreased overall (24). Which was in accord with the theory of a physiological role for leptin in the regulation of human sperm motility (25). Excessive apoptosis-mediated sperm cell death might be an important cause of reduced sperm density, which consequently leads to reduced male fertility or even infertility (26). It is known that varicocele induces the production of metabolic toxins such as reactive oxygen species (ROS) (27, 28). Studies have shown that seminal plasma leptin may be involved in the process of ROS-mediated excessive apoptosis of sperm cells (28, 29). Meanwhile, the quality of spermatogenesis is related to the level of testosterone secretion (14). Overexpression of leptin receptors and reduced testosterone secretion in the testes of patients with varicocele may also be a mechanism that contributes to reproductive disorders (13, 30).

In fact, the exact mechanism linking leptin to reproductive function in patients with varicocele remains to be determined. Hypoxia is thought to be one of the most critical pathological mechanisms of spermatogenic dysfunction associated with varicocele (31–33). HIF-1a, a master regulator of the response to hypoxia (34), was detected with increased expression in the internal spermatic veins of patients with varicocele (31, 35). It has been reported that the leptin and HIF-1α mRNA correlated positively under hypoxia (20). This implies that leptin may be involved in the HIF-1α-related hypoxic response and result in spermatogenic dysfunction. Additionally, increase of leptin levels in the seminal plasma of patients with varicocele can induce oxidative stress (e.g., increased expression of ROS and TNF-a), leading to an increased rate of sperm apoptosis and consequent functional defects of sperm (13, 28, 29).

### Limitations

However, our study has some limitations. Firstly, the number of available evidence is limited. More studies with larger sample sizes would make the results more convincing. Secondly, studies exploring the relationship between leptin and sperm parameters are limited, and more relevant studies would be helpful to discover the relationship between leptin and sperm parameters. Finally, considering the limited numbers of included studies, the publication bias was not conducted, which indicated potential bias existed.

## Conclusions

The expression of leptin was significantly increased in the seminal plasma of infertile patients with varicocele, which was negatively correlated with sperm concentration and progressive motility. Although the pathophysiological role of leptin in patients with varicocele is unclear, it is a promising potential biomarker and therapeutic target for fertility dysfunction in patients with varicocele, given its effects on sperm. More studies exploring the effects of leptin on reproductive function in patients with varicocele are needed.

## Supplementary material

Supplementary Figure 1 Forest plot of the sensitivity of the summary Pearson correlation coefficients. **(A)** for seminal leptin level and concentration of sperm, **(B)** for seminal leptin level and progressive motility of sperm.

Supplementary Table 1 Results of quality assessment of included studies.
