# Supplementary figures and images for: The role of leptin in reproductive dysfunction in patients with varicocele: a systematic review and meta-analysis
Source: Front Urol. 2026 Jun 5;6:1835856. doi: 10.3389/fruro.2026.1835856 (PMC13278944; doi:10.3389/fruro.2026.1835856)

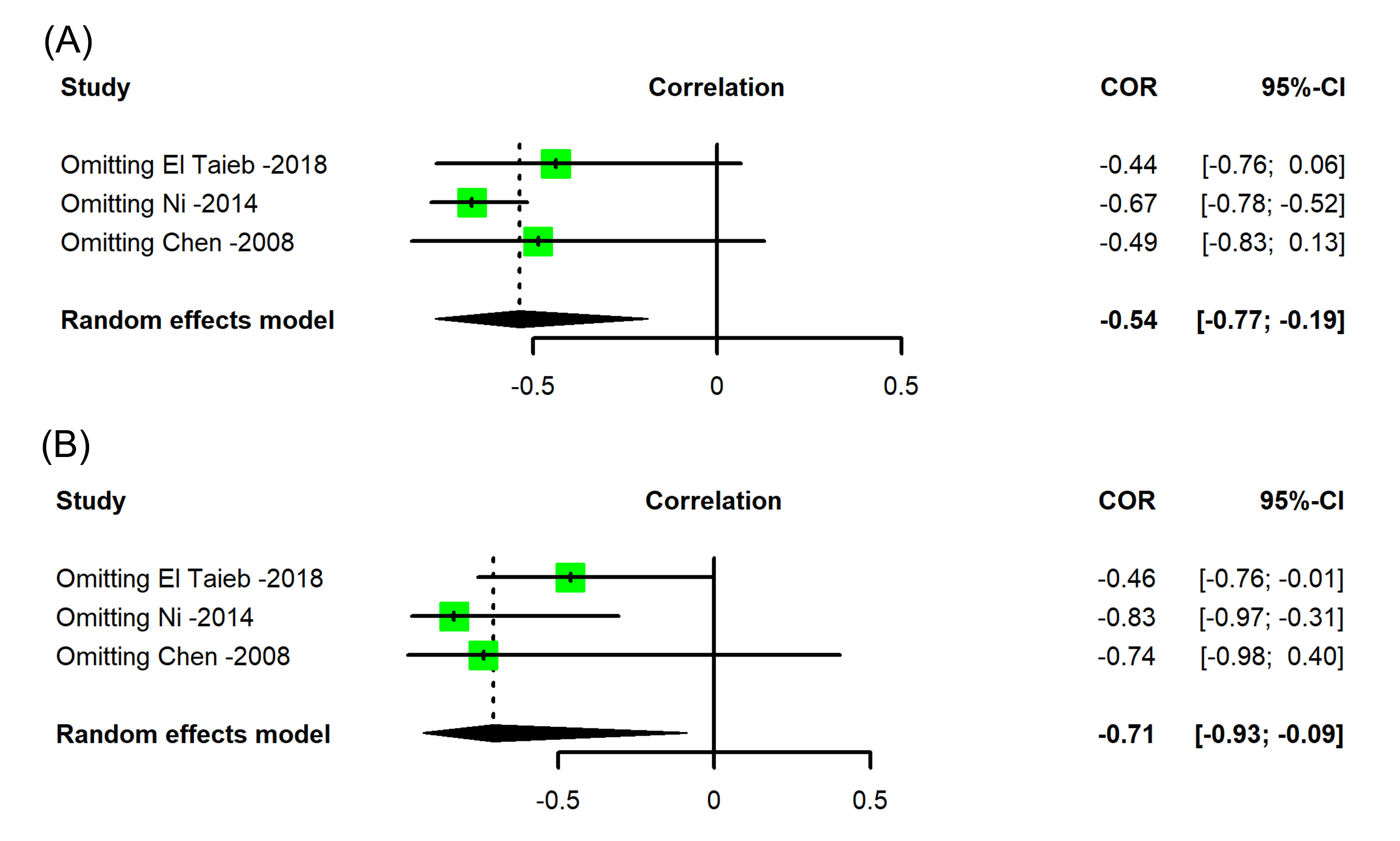

Supplement: Supplementary Figure 1 — Forest plot of the sensitivity of the summary Pearson correlation coefficients. (A) for seminal leptin level and concentration of sperm, (B) for seminal leptin level and progressive motility of sperm. [file Image1.tif]
